# Clonality of HTLV-2 in natural infection

**DOI:** 10.1186/1742-4690-11-S1-P138

**Published:** 2014-01-07

**Authors:** Anat Melamed, Aviva D Witkover, Rachael Brown, Kristin Ladell, Niall Gormley, Edward L Murphy, Graham P Taylor, David A Price, Charles RM Bangham

**Affiliations:** 1 Section of Immunology, Imperial College London, Wright-Fleming Institute, Norfolk Place, London, UK; 2 Institute of Infection and Immunity, Cardiff University School of Medicine, Cardiff, Wales, UK; 3 Illumina, Chesterford Research Park, Essex, Little Chesterford, UK; 4 University of California San Francisco, California, USA; 5 Section of Infectious Diseases, Imperial College London, Wright-Fleming Institute, Norfolk Place, London, UK

## 

We recently developed a high-throughput sequencing method for analysis and quantification of HTLV-1 integration sites in the host genome (Gillet et al, 2011, Blood). Using this method we investigated the effect of the genomic environment on integration targeting, clonal expansion and spontaneous HTLV-1 proviral expression (Gillet et al, 2011, Blood, Melamed et al, 2013, PLoS Pathogens). HTLV-2 preferentially infects CD8+ T cells, with a minority of the proviral load in CD4+ T cells. Here we describe the use of our high-throughput technique to investigate the distribution of HTLV-2 proviral integration sites in the host genome, in peripheral blood mononuclear cell (PBMC) DNA of HTLV-2 infected individuals (n=28). We also mapped and quantified proviral integration sites separately in flow-sorted CD4+CD8- and CD4-CD8+ populations. We quantified the clone frequency distribution and clonal survival over time in 10 individuals, using samples from 2 time points separated by a median of 10 years. The results show that the clone frequency distribution of HTLV-2 in PBMCs is distinct from that of HTLV-1 and resembles that of HTLV-1-infected CD8+ T cells. These results suggest that in both HTLV-1 and HTLV-2 infections, there is a greater degree of selective oligoclonal clonal expansion in infected CD8+ T cells than in CD4+ T cells. We are now investigating the selection forces that underlie this dichotomy between T cell lineages.

